# Meaningful connections in dementia end of life care in long term care homes

**DOI:** 10.1186/s12888-018-1882-9

**Published:** 2018-09-24

**Authors:** Lynn McCleary, Genevieve N Thompson, Lorraine Venturato, Abigail Wickson-Griffiths, Paulette Hunter, Tamara Sussman, Sharon Kaasalainen

**Affiliations:** 1 Department of Nursing, Brock University, St. Catharines, Canada; 2 College of Nursing, University of Manitoba, Winnipeg, Canada; 3 Faculty of Nursing, University of Calgary, Calgary, Canada; 4 Faculty of Nursing, University of Regina, Regina, Canada; 5 Department of Psychology, St. Thomas More College, University of Saskatchewan, Saskatoon, Canada; 6 School of Social Work, McGill University, Montreal, Canada; 7 School of Nursing, McMaster University, Hamilton, Canada

**Keywords:** Palliative care, Nursing homes, Death, Bereavement, Illness trajectory

## Abstract

**Background:**

Most persons with dementia die in long term care (LTC) homes, where palliative approaches are appropriate. However, palliative approaches have not been widely implemented and there is limited understanding of staff and family experiences of dying and bereavement in this context.

**Method:**

This descriptive qualitative study explored family and staff experiences of end of life and end of life care for persons with dementia in LTC homes. Eighteen focus groups were conducted with 77 staff members and 19 relatives of persons with dementia at four LTC homes in four Canadian provinces.

**Results:**

Three themes emerged: *knowing the resident*, the understanding that *they are all human beings*, and the *long slow decline and death* of residents with dementia.

**Discussion:**

Intimate knowledge of the person with dementia, obtained through longstanding relationships, was foundational for person-centred end of life care. Health care aides need to be included in end of life care planning to take advantage of their knowledge of residents with dementia. There were unmet bereavement support needs among staff, particularly health care aides. Persons with dementia were affected by death around them and existing rituals for marking deaths in LTC homes may not fit their needs. Staff were uncomfortable answering relatives’ questions about end of life.

**Conclusions:**

Longstanding intimate relationships enhanced end of life care but left health care aides with unmet bereavement support needs. Staff in LTC homes should be supported to answer questions about the trajectory of decline of dementia and death. Further research about residents’ experiences of deaths of other residents is needed.

## Background

The prevalence of dementia is increasing dramatically with population aging. Dementia, a class of neurodegenrative diseases, is characterized by progressive deterioration and decline in cognition and functioning. In late stages of dementia, the person is completely dependent on care providers. Although community-based care for dementia is more common than it once was, in North America most people with dementia die in long term care (LTC) homes [1].

Lack of access to palliative care is a problem for persons with dementia, who are less likely than people with cancer to be referred to palliative care despite a similar burden of suffering [2]. In recent years, increased attention has been paid to the need for better end of life and palliative care for persons with dementia. The European Association for Palliative Care (EAPC) white paper on palliative care for persons with dementia affirmed that because dementia is a terminal condition, with care focused on maintaining and improving quality of life, palliative care is appropriate for dementia [3].

A palliative approach includes attending to comfort needs, psychosocial needs, spiritual needs, symptom management, and advanced care planning. The focus is on quality of life of the person with dementia and their family or informal carers [4]. It is congruent with the person centred approach of good dementia care [5, 6].

Literature on end of life care and dementia in LTC is emerging, particularly in the areas of advance care planning [7, 8] and pain and symptom management [2, 9], with less research about psychosocial and spiritual aspects of palliative care.

There is some evidence of suboptimal palliative care for persons with dementia in LTC homes [2, 10]. In their synthesis of evaluations of programs to improve palliative care in LTC homes, Goodman and colleagues concluded that “for people with dementia living and dying in care homes uncertainty is an inevitable and integral part of end of life care” [27, 11]. Uncertainty means that it is difficult to predict death or recognize when someone with dementia is nearing end of life. Additional challenges for staff and families include lack of recognition of dementia as a life-limiting illness and the high prevalence of comorbid conditions in end stage dementia [2, 11, 12].

Emerging evidence indicates that comprehensive programs that include staff training and tools to support practice change can improve end of life in LTC homes [11, 13, 14]. This literature also highlights the importance of a holistic palliative approach that attends to the psychosocial and spiritual needs of staff and families as the resident dies and in their bereavement [15]. Better understanding of staff and family experiences of end of life and end of life care of persons with dementia in LTC homes is essential for implementing holistic palliative care programs. The purpose of this study was to describe family and staff experiences of end of life and end of life care of persons with dementia in LTC homes. Specifically, we explored how care near the end of life is provided, what facilitates this care, and challenges experienced by staff and families.

## Method

This descriptive qualitative study was conducted as a sub study during baseline data collection for a multi-site investigation of a program to enhance end of life care in LTC homes [16]. The research was approved by five university Research Ethics Boards. The study was conducted in four LTC homes in four Canadian provinces. The homes have between 50 and 284 beds and between 14 and 58 deaths per year. Two have segregated dementia units.

Multiple focus groups were conducted at each LTC home. Four focus groups were conducted with relatives of residents of the LTC homes, one at each site. Family participants were recruited through a combination of flyers, email letters, posters, and through the Family Councils of the LTC homes. Thirteen staff focus groups were held (3 or 4 in each LTC home). Staff were recruited through information sessions at staff meetings and posters in the LTC homes inviting staff to participate. All participants provided written informed consent.

Focus groups lasted between 30 min and about an hour and were audiorecorded and transcribed verbatim. The interview guides prompted discussion about end of life practices in the LTC home; compassion and comfort in end of life care; staff comfort with providing end of life care, including barriers, facilitators, and learning needs; and family expectations of end of life care. Staff focus groups included discussion of experiences talking about end of life with residents and families, while family focus groups discussed experiences talking with staff about end of life. Participants were asked about the impact of dementia as the various topics were discussed.

Thematic analysis [17] was conducted beginning with reading and rereading the transcripts and preliminary discussion of the data (LM, GT, LV). Initial data coding, collating codes into emergent themes, and comparison of findings within and between staff and family focus group transcripts, was conducted by the first author, followed by discussion of and confirmation of themes and discussion of the meaning of findings by three authors (LM, GT, LV).

## Results

### Sample

Between 4 and 5 family members attended each family focus group, resulting in 19 family participants. Their relatives had been living in the LTC home for an average of 4.5 years (SD = 4.5; range, less than 1 to 13 years). A description of the family member sample is presented in Table 1.

**Table 1:** Family participant characteristics (*N* = 19)

Characteristic	N	%
Sex		
Female	14	73.7
Male	5	26.3
Kin relationship to resident		
Child	14	73.7
Spouse	2	10.5
Sibling	1	5.3
Other	2	10.5
Age in years		
45 to 54	1	5.3
55 to 64	8	42.1
65 to 74	7	36.8
75 to 84	2	10.5
85 or older	1	5.3

Between 3 and 13 staff members attended each staff focus group, resulting in 77 staff participants. The average number of years employed at the LTC home was 8.9 (SD = 7.8; range, less than 1 to 38 years). A description of the staff sample is presented in Table 2.

**Table 2:** Staff participant characteristics (*N* = 77)

Characteristic	N	%
Sex		
Female	69	89.6
Male	7	9.1
Missing	1	1.3
Age		
Under 25	2	2.6
25 to 34	11	14.3
35 to 44	17	22.1
45 to 54	24	31.2
55 to 64	16	20.8
65 and older	7	9.1
Profession		
Health care aide	26	33.8
Nurse (registered nurse, registered practical nurse, licensed practical nurse)	22	28.6
Allied Health Professional	13	16.9
Housekeeping and Dietary Staff	8	10.4
Manager	5	6.5
Support Staff	3	3.9
Employment Status		
Full time	56	72.7
Part time	18	23.4
No response	3	3.9
Completed Some Palliative Education or Training		
Yes	47	61.0
No	26	33.8
No response	4	5.2

### Themes

Three themes emerged: (1) the importance of knowing the resident; (2) they are all human beings; and (3) long slow decline and death in dementia. Following is an elaboration of each theme and the sub-themes within them.

### Knowing the resident

That LTC staff have an intimate knowledge of the person with dementia emerged as significant to both staff and family participants. This theme reflects the importance to participants that staff know the person with dementia intimately. Knowing the person with dementia allowed staff to provide comfort and compassion when the person with dementia could not communicate through words. Knowing the person with dementia was a result of the long time that staff spent working with the person. Staff thought that when persons with dementia were admitted within a few months of their eventual death, staff did not have enough time to get to know the person, making it more difficult to provide person-centred end of life care. There were three sub-themes within this theme: time for staff to come to know the resident intimately; time to establish a relationship; and consistent staffing.

#### Time to know the resident intimately

As a result of time spent caring for the person with dementia, health care aides, dietary aides, and activation recreation staff knew the person with dementia intimately. They knew their routines, their preferences for food and beverages, their facial expressions, what position they preferred for sleeping, and what comforted them. This intimate knowledge made it easier to provide comfort and to overcome communication challenges with persons with dementia, especially when the dying person was unresponsive either because of being in late stages of dementia or because death was near. Knowing the person intimately was important to staff and to families. As one son described:

You have to get to know them personally… and obviously as you get to know that person, then I think you can provide that compassionate care because you know what their needs are. (Family ID 1)

Knowing the resident intimately allowed staff greater ability to detect subtle changes, particularly in regard to pain. Providing adequate pain management at the end of life was critically important to staff and family participants. Staff reported that it was difficult to assess pain of persons with dementia. Health care aides and family participants thought that knowledge of the person with dementia contributed positively to pain assessment and management. A health care aide described:

Even the dementia residents do not tell you they are in pain, you can see it in their face that they are in pain. (Staff ID 14)

#### Time to establish relationships

This theme emerged primarily from descriptions provided by families and health care aides, as well as from supervisors’ descriptions and observations about health care aides, dietary aides, and activity and recreation staff. For health care aides, working with a resident with dementia over many months or even years meant that they had established close relationships. Health care aides viewed themselves as an extension of the resident’s family and described their relationships as family-like or friendships. For some, these relationships made it easier to be compassionate at the end of life. Health care aides described that the person with dementia reacts differently to them during end of life, depending on the relationship that is established and the quality of their relationship. These close relationships were reported to result in persons with dementia, even when mostly unresponsive, being able to recognize the staff member’s presence and be comforted by it. Two health care aides discussed this:

First health care aide: I work on the dementia units. Most of our seniors when they get to the point when they are dying, they don’t talk much. They don’t really know much about what’s going on. But, yet, you do see some responses to being there with them. (Staff ID 20)

Interviewer: Yeah.

Second health care aide: Whether it’s they relax more, or they will cling to your hand, or start mumbling, or kind of sing quietly. (Staff ID 21)

These longstanding close relationships also meant that the loss of the relationship when the resident died could be felt deeply. Health care aides spoke about experiencing sadness, loss, and grief when residents with dementia die. They discussed the importance of maintaining relationships after a resident or health care aide was transferred to another unit, especially being able to visit before the resident died. A health care aide explained:

It’s kind of sad when you suddenly, you didn’t even have a visit. So, if you have a last visit on their last day, you say goodbye. It feels okay. (Staff ID 18)

Staff experiences of grief and need for time to mourn were recognized by family participants and by supervisor participants. However, some health care aides felt unsupported by their supervisors, who did not acknowledge the meaning of the death and loss for them. For some health care aides, it was very important to attend a resident’s funeral or funeral home visitation, especially if they felt particularly close to the resident. It was uncommon for them to be provided with time to attend funerals or visitations.

#### Consistent staffing

For both family and staff participants, having time to know the person with dementia and time to establish relationships was linked to consistent staffing. Families thought that staff consistently working on the same units made it possible for staff to attend to and notice signs of distress or agitation that might indicate pain or fear. Staff and managers who worked in a LTC home where staff did not rotate between units thought that this staffing practice was important both for achieving intimate knowledge of their residents and for residents being comfortable with staff. A manager explained:

Here you come in say year one and group one (of residents) and year ten you are in group one. They always have, and that’s really helpful with dementia because they get to recognize them. (Staff ID 23)

### They are all human beings

This theme reflects staff and family views about what they referred to as the “human” experience of end of life and grief for persons with dementia. There were three sub-themes; the first was persons with dementia deserving good end of life care; the second, person centred end of life care for residents with dementia; and the third, living with death around them.

#### Persons with dementia deserve good end of life care

When asked about differences in providing end of life care for persons with dementia compared to residents who do not have dementia, staff were consistently very quick to respond that it does not matter whether the dying person has dementia. This seemed to be related to staff values for equity and good end of life care; that everyone deserves comfort and compassion at the end of their life. Staff often referred to the dementia diagnosis not defining the person, that people with dementia are “still human” or “all human beings.” Family participants expressed hope that end of life care would be similar for those with and without dementia.

#### Providing person centred end of life care for residents with dementia

In further discussion, staff and family described some unique aspects of end of life care for persons with dementia, as compared to residents who do not have dementia. These related to time and effort to provide nursing care, the impact of behavioral symptoms of dementia, and the importance of touch for persons with dementia.

##### Time and effort

Staff explained that care takes more time when someone is dying, even more so when the person has dementia and cannot express their needs verbally. Nurses and health care aides referred to needing to use their knowledge of the person and go through more of “a process of figuring out what comforts them” (Staff ID 67) when the dying person has dementia. A health care aide discussed and explained the challenge of positioning a resident and the importance of teamwork:

Two person [care] is good, especially the dementia person. When they are dying it is very hard for us. For resident also it’s very hard. So, if two person, we can consult each other to put position that would be best for her. And always we keep an eye on how he or she is doing. (Staff ID 16)

Extra time and effort was also needed for communication with and reassuring the person with dementia at end of life. A health care aide explained:

If they are palliative, until they are unresponsive, sometimes just knowing that you might have to repeat things so many times to them about their condition and what you are going to do …. It seems like you have to give even more reassurance and repeat yourself more often to answer their questions more frequently because they do tend to forget what you say to them or maybe respond in a different manner than someone else might, who does not have dementia. (Staff ID 65)

Family participants also thought that end of life care would take more time with a resident who had dementia. Time that, in many cases, they thought staff did not have.

##### Behavioural symptoms

Staff and family participants explained that if the person with dementia has “aggressive” behaviours, then it is more challenging for staff to be compassionate. As a health care aide explained:

Depends on what behaviours they have, unfortunately. If somebody comes in and they are aggressive with us from day one until the day they pass away, like, I don’t particularly like getting physically hit. It makes it harder to spend that quality time with them. (Staff ID 20)

A daughter expressed similar thoughts about staff interactions with residents who have behavioural symptoms:

If they don’t have training or understanding how to deal with it, most people, just by human nature, I think, are going to try and back off. (Staff ID 5)

##### Touch

For staff and family participants, touch was described as an important way of demonstrating compassion and care for anyone who is dying, especially for persons with dementia. A manager explained:

Touch can sometimes be that final way of connecting when the words don’t make sense any more. Or I can’t even see you or if I’m seeing this cup and I don’t even know what it is. Just the presence and touching. (Staff ID 25)

#### Living with death around them

Family and staff participants described persons with dementia noticing deaths of other residents, being affected by the deaths, and experiencing grief. Family participants described LTC home residents as living with death around them. There were differing opinions among family participants about how to acknowledge deaths. Some thought it might not be good to tell their relatives that someone had died, others thought that the residents with dementia needed support when someone in the LTC home died. A son described his father noticing and being affected by the death of a resident he did not know well:

Well, you know, he doesn’t even know the fellow. But, you know, he’s only been here a few days and hasn’t even met the fellow. But, you know, just knowing this is kind of his last home. It bothered him. (Family ID 6)

A daughter, discussing what it was like when a roommate died, recounted:

Even if the roommate had dementia, you know, they’re still… you can still get little flashes of insight … having someone die in your room would be very upsetting. Because my Dad did have a roommate die. He was aware of it. (Staff ID 17)

According to staff participants, persons with dementia know when someone dies, they feel the loss, need support, and, for the most part, are not supported in their grief. As one staff participant explained:

You remember when (resident’s name) died and (resident’s name) got so upset. So, the residents build relationships with each other, right. They get close. But when you have dementia, you still have closeness. (Staff ID 15)

Several of the homes recognized deaths of residents by posting memorial lists of names or photographs of residents who died. This was not viewed as sufficient support for residents with dementia. As described by a staff participant:

There is very little support for the roommate or the friends of the person who has passed away. Like, it’s all informal, right? (Staff ID 20)

One home had a practice of recognizing a resident’s death by putting a rose at the person’s place in the dining room at a meal the day after the person dies. At the meal, there would be a brief acknowledgement of the person’s death and a prayer. This practice was seen by family participants as a good way to recognize and support their relatives’ grieving.

### Long slow decline and death

This theme reflects staff and family views about the long slow decline and death of the person with dementia. There were three sub-themes in this theme: prolonged losses; uncertainty for families about the dementia illness trajectory; and death as a release from misery.

#### Prolonged losses and decline

Family participants talked about the prolonged suffering of their relatives and the multiple losses they experienced. They viewed the LTC home as the “end of the line,” as one daughter described it (Family ID 7). Another family participant explained: “People in long term care are gradually dying very slowly” (Family ID 18).

There were varying opinions about what this means for families when their relative dies. Some family participants thought that compared to death of a relative from another cause, the prolonged losses and decline experienced with dementia might make death worse for families. A daughter anticipated that her grief may be easier than grief associated with death from other causes:

We lived with my Mom in long term care for so many years and watched the slow decline with dementia. It’s like we… we’ve started saying goodbye a long time ago, so the final breath isn’t, won’t be as traumatic as maybe a sudden death would be. (Family ID 15)

Some staff and family participants referred to persons with dementia as “already lost” (Staff ID 23), “not being the same person” (Family ID 6), or that “they are not really alive the way they should be” (Family ID 8) by the time late stages of dementia and end of life occurred. This was more common among registered nursing staff than health care aides, who also reported closer relationships with residents with dementia. This perceived loss of the person to dementia prior to death, was seen as resulting in some family members not being there when the person with dementia died. A recreation therapist described an experience:

I have sat with a resident while he was passing away as long as I could before. Just because the family had said. It wasn’t here. The family has said that I lost my father years ago, I don’t need to be here. (Staff ID 75)

#### Family uncertainty about the dementia illness trajectory

While staff participants thought that some families viewed their relative as already lost or gone, they also found that families are often not ready for their relative’s decline and uncertain about the trajectory of decline associated with dementia. Staff thought that understanding this decline would help families anticipate and accept death. A nurse explained:

We have people with dementia, families are expecting them to be behaving and acting the same way they used to six months ago or even sometimes, when they were at home. And there are slow, gradual changes. And they are thinking, well the individual can’t hear. That is what the problem is. The problem is not that the hearing has deteriorated, it is that the information processing has changed and they are not ready to accept that. They want the hearing issue dealt with. (Staff ID 66)

Similarly, family participants described how important it was to understand dementia and the course of illness. They needed answers to their questions about how much longer their relative would live. They wanted to know what death would be like and what kind of care their relative would receive as death neared. For some, there was a perception that staff were not comfortable with these discussions. As described by a sibling of a resident:

To me, helping people understand, really, really understand what an end of life process is. It just. Then there still seems to be an incredible amount of reluctance on the provider’s side to even talk about that. Because it’s not necessarily a pretty process. It seems like the general belief is it’s better for people to be surprised by it or whatever. I don’t know what the hell it is but and because of that people don’t properly prepare. (Family ID 10)

Staff participants explained that it was difficult to answer families’ questions about resident comfort, how long the person had to live, or when death could be expected. A health care aide described being “totally outside my comfort zone” (Staff ID 21) when interacting with families of residents with dementia who are dying. A nurse described being comfortable with families, attributing this to interacting with and coming to know families over time on a dementia unit.

#### Death is a release from misery

Family participants described the long process of decline as suffering. Many staff viewed death of a person with dementia as a release from suffering and misery. This suffering included the losses that the person experienced over the course of dementia and their experiences of behavioural and psychological symptoms of dementia. A health care aide described the relief she felt when someone died:

You feel sorry for them. The aggressive person. … But it’s good they can go peacefully. (Staff ID 28)

Staff participants reported that families were usually, but not always, grateful that their relative had been released from suffering.

A health care aide explained:

I think it is kind of more comforting for the family, though. Like, if … their death had been caused by the dementia. You know what I mean. They had just been continually declining and it is almost, they feel like they are not suffering anymore … sometimes I think family are happy to know that they are in a better place. (Staff ID 73)

Some staff thought that their relative’s death might be a relief from stress for families but that some families were not ready to let go. A nurse explained:

I think sometimes family members are more, not in every case, but family members are more willing to let go and to almost. There is almost a positive anticipation. Not sure if you would agree. But, sometimes I guess there is a lot of stress and challenges when a family member is suffering dementia and, so, maybe that arises out of a sense of there being relief. (Staff ID 77)

## Discussion

Close intimate relationships between staff and residents were seen as vital for providing end of life care and overcoming challenges related to the person with dementia’s diminished communication ability. These findings have implications for implementing palliative approaches to end of life care for persons with dementia in LTC homes. The person centred care approach that is advocated in dementia care continued to be relevant at end of life. Relationships, valuing people with dementia, and treating people as individuals are core tenants of person centred care [18] and were evident in the *knowing the resident* and *they are all human beings* themes. This is consistent with the principles of palliative care and the European Association for Palliative Care (EAPC) white paper on dementia [3]. However, there are varying opinions about the value of a palliative approach in advanced dementia care [4]. Efforts to adopt palliative care approaches should build on commonalities between good palliative and end of life care and the person centred care approaches that are already used in LTC homes [5, 6]. When implementing palliative care with persons with dementia in LTC homes, training and education can help staff to see the link between end of life care practices and the person centred care approaches they value and are already using.

Pain management is notoriously challenging with persons with dementia, particularly at the end of life [2]. Pain and agitation are common among persons with dementia at the end of life, and pain interventions are underutilized [2, 9, 19]. Staff and families thought that intimate knowledge of the person with dementia made it easier to know when a dying person with dementia was in pain and respond to signs of pain, agitation, or distress. Health care aides were confident about recognizing pain. The close relationship between the health care aides and residents, the health care aides’ intimate knowledge of the person, and their ability to recognize subtle indicators of discomfort and distress should be leveraged to improve pain management. However, inadequate acknowledgement and support of health care aides’ ability to detect pain and advocate for residents is a barrier to improving pain management in LTC [20]. Health care aides’ opinions are frequently dismissed, and they are not included in case conferences where pain management is discussed [21]. Implementing comprehensive palliative care programs such as the Gold Standard Framework in Care Homes can result in improved collaboration between nursing staff and health care aides [13], potentially paving the way to incorporating health care aides’ intimate knowledge of dying residents with dementia in their pain management.

The amount of time that a person with dementia lives in a LTC home varies by jurisdiction from months to several years [22]. In Canada length of stay at time of death in LTC homes is decreasing [23]. This trend may impact end of life care for residents with dementia. In this study, staff indicated that when there was a shorter length of stay, they did not feel the same connection to and knowledge of the needs, preferences and essence of who the dying resident is.

The advantages that close relationships and intimate knowledge bestow on end of life care come at a cost for staff, especially for health care aides. This is consistent with previous research where staff described family-like relationships and grief associated with end of life care of persons with dementia [24, 25] and points to the importance of supporting staff grief and bereavement [15]. However, previous research has identified issues of inadequate support for staff related to grief and emotional needs [24–27]. The close relationships between staff and residents with dementia are not acknowledged when residents die and there is not enough time for staff to grieve [25]. Similarly, health care aides in a previous study received support in their grief from their peers but not from supervisors [27].

Administrators and supervisors recognized the need to support staff after residents died. Managers’ concern about staff wellbeing after residents die supports feasibility of increasing support from supervisors. End of life education and support needs vary depending on staff role [28]. Supervisors who are aware that health care aides may be less likely than other staff to view the person with dementia as “already gone” prior to death may be better able to support their grief and purposefully check in with and acknowledge grief when a resident with dementia dies.

Supporting staff as residents deteriorate and after their deaths is an important part of end of life dementia care [15]. Some LTC homes had rituals to acknowledge the death of a resident; rituals that could play a role in acknowledging and supporting staff grief, such as memorial photographs, having a staff honor guard when the resident’s body leaves the building, and memorial services. These practices and others, such as providing emotional support through regular discussions about recent deaths, have been identified as positive [29, 30]. However, for some staff, rituals in the LTC home were insufficient and it was important to be able to attend the deceased resident’s funeral or visitation. This was rarely possible. While resources are limited in LTC homes, supporting staff grief and need to participate in funeral rituals would be consistent with and demonstrate value for the relationships staff form with residents with dementia.

Several writers have discussed death as “hidden” in LTC homes [27, 31]. This aspect of the culture of LTC homes is relevant to our findings about the impact of death on surviving residents with dementia in LTC homes and family need for information about the illness trajectory of dementia. Consistent with recent research, staff and family participants identified unmet support needs for residents with dementia when a fellow resident died [32]. Posting a memorial name or photograph was seen as inadequate, especially for residents with dementia. The ceremonial marking of a resident’s death by placing a rose at their dining table and praying at a meal shortly after the resident died seemed to be meaningful to staff and seen as positive for residents. Long term care homes should consider adapting and implementing this practice.

Helping families to understand the trajectory of decline and death in dementia is important in LTC homes. Family recognition of dementia as a terminal illness is associated with better resident comfort at end of life [33]. Family participants seemed to understand dementia as a terminal illness. However, they did not understand the trajectory of decline of dementia and some wanted to know what death would be like. Relatives of persons with moderate to severe dementia living in LTC homes have been found to misunderstand the terminal nature of dementia, the trajectory of decline, and what death would be like, even after the disease process has been explained to them [31, 34]. In recent research, bereaved relatives indicated that they would have appreciated receiving verbal and written information about the course of dementia and end of life [35]. Thus, there is a need to provide information in a variety of formats and repeatedly, and for staff to convey openness to answering questions about decline and death.

Some family participants perceived staff discomfort with answering [8] questions about death and the illness trajectory, consistent with previous findings [36]. These perceptions are likely accurate; staff reported being uncomfortable answering families’ questions about decline and death. Health care providers’ limited knowledge of the death process and the dementia illness trajectory have been identified in the literature [37] and LTC home staff have attributed their reluctance to discuss end of life with relatives of persons with dementia to lack of confidence [26]. Additionally, in LTC homes, uncertainty about when death is imminent for residents with advanced dementia is common [11]. Together, these findings support a need to provide staff with education about the trajectory of dementia, the challenges of recognizing when someone is dying, and the death process.

Limited skills for discussing emotion-laden topics or limited understanding of the therapeutic value of such discussions may also play a role in staff discomfort answering families’ questions and discussing death. Staff may benefit from knowing that coming to understand the dementia illness trajectory relieves families [38]. End of life education for health care workers should include communication skills training and practice talking to relatives about decline and death of a person with dementia. Understanding that grief and distress are common for family of persons in the late stages of dementia [15, 35] may enhance staff’s ability to respond to families. There is some evidence that such approaches have been effective in palliative care settings [39].

Methodological strengths of this study include the number of focus groups and diversity in the sample with respect to the LTC homes and the staff participants. LTC homes were purposefully sampled to include for-profit and not-for-profit homes, in different provinces, which vary in terms of funding and regulations for LTC homes. Diversity in the staff sample with respect to professional designation and job category meant that various staff perspectives and experiences were included. The inclusion of family and staff participants allowed us to compare their perspectives. The sample may be biased towards people who strongly value end of life care and this may be associated with some characteristics that were reported to be associated with experiences and challenges in the provision of end of life care.

The findings suggest opportunities to support end of life care for persons with dementia in LTC homes. It is important to acknowledge and support the value of relationships, the time it takes to establish relationships, and the grief experienced by staff who provide care. Health care aides’ intimate knowledge of persons with dementia should be incorporated in interprofessional pain management interventions. Residents with dementia are not immune to grief and loss when other residents die. Further research about their needs is required. Existing rituals within LTC homes may need to be enhanced. Families want and need information about the dementia illness trajectory and end of life. Education and training for staff to be confident in providing this information and answering families’ questions is needed.

## Abbreviations

EAPC: European Association for Palliative Care
LTC: Long term care

## References

[1] Reyniers T, Deliens L, Pasman HR, Morin L, Addington-Hall J, Frova L, Houttekier D (2015). International variation in place of death of older people who died from dementia in 12 European and non-European countries. JAMDA.

[2] Dempsey L, Dowling M, Larkin P, Murphy K (2015). The unmet palliative care needs of those dying with dementia. Int J Palliat Nurs.

[3] van der Steen JT, Radbruch L, Hertogh CMPM, de Boer ME, Hughes JC, Larkin P…, Volicer L (2014). White paper defining optimal palliative care in older people with dementia: a Delphi study and recommendations from the European Association for Palliative Care. Palliative Med.

[4] Sampson E, van den Noortgate N, van der Steen JT, Gibson SJ, Lautenbacher S (2017). Palliative care of patients with dementia and pain. Pain in Dementia.

[5] Hughes JC, Jolley D, Jordan A, Sampson EL (2007). Palliative care in dementia: issues and evidence. Adv Psychiatr Treat.

[6] Kydd A, Sharp B (2016). Palliative care and dementia – a time and place?. Maturitas.

[7] Beck E, McIlfatrick S, Hasson F, Leavey G (2017). Health care professionals’ perspectives of advance care planning for people with dementia living in long-term care settings: a narrative review of the literature. Dementia.

[8] Jones K, Birchley G, Huxtable R, Clare L, Walter T & Dixon J. End of life care: a scoping review of experiences of advance care planning for people with dementia. Dementia 2016; 0;1–21. 10.1177/1471301216676121. 10.1177/1471301216676121 27821714

[9] Hendriks SA, Smalbrugge M, Galindo-Garre F, Hertogh CMPM, van der Steen JT (2015). From admission to death: prevalence and course of pain, agitation, and shortness of breath, and treatment of these symptoms in nursing home residents with dementia. JAMDA.

[10] Mitchell SL, Kiely DK, Hamel MB (2004). Dying with advanced dementia in the nursing home. Arch Intern Med.

[11] Goodman C, Froggatt K, Amador S, Mathie E, Mayrhofer A (2015). End of life care interventions for people with dementia in care homes: addressing uncertainty within a framework for service delivery and evaluation. BMC Palliat Care.

[12] Hill SR, Mason H, Poole M, Vale L, Robinson L (2017). What is important at the end of life for people with dementia? The views of people with dementia and their care partners. Int J Geriatr Psych..

[13] Badger F, Plumridge G, Hewiston A, Shaw K, Thomas K, Clifford C (2012). An evaluation of the impact of the gold standards framework on collaboration in end-of-life care in nursing homes. A qualitative and quantitative evaluation. Int J Nurs Stud.

[14] Kinley J, Stone L, Dewey M, Levy J, Stewart R, McCrone P, Sykes N, Hansford P, Begum A, Hockley J (2014). The effect of using high facilitation when implementing the gold standards framework in care homes programme: a cluster randomised controlled trial. Palliat Med.

[15] Jones L, Candy B, Davis S, Elliott M, Gola A, Harrington J, Kupeli N, Lord K, Moore K, Scott S, Vickerstaff V, Omar R, King M, Leavey G, Nazareth I, Sampson E (2016). Development of a model for integrated care at the end of life in advanced dementia: a whole systems UK-wide approach. Palliat Med.

[16] Kaasalainen S, Sussman T, Neves P, Papaioannou A (2016). Strengthening a palliative approach in long-term care (SPA-LTC): a new program to improve quality of living and dying for residents and their family members. J Am Med Dir Assoc.

[17] Braun V, Clarke V (2006). Using thematic analysis in psychology. Qual Res Psychol.

[18] Hunter PV, Hadjistavropoulos T, Kaasalainen S (2015). A qualitative study of nursing assistants’ awareness of person-centred approaches to dementia care. Ageing Soc.

[19] Hendriks SA, Smalbrugge M, Hertogh CMPM, van der Steen JT (2014). Dying with dementia: symptoms, treatment, and quality of life in the last week of life. J Pain Symptom Manag.

[20] Kaasalainen S, Brazil K, Coker E, Ploeg J, Martin-Misener R, Donald F, Burns T (2010). An action-based approach to improving pain management in long-term care. Can J Aging.

[21] Jansen BDW, Brazil K, Passmore P, Buchanan H, Maxwell D, McIlfatrick SJ, Parsons C (2017). Exploring healthcare assistants’ role and experience in pain assessment and management for people with advanced dementia towards the end of life: a qualitative study. BMC Palliat Care..

[22] Kelly A, Conell-Price J, Covinsky K, Cenzer IS, Chang A, Boscardin WJ, Smith AK (2010). Lengths of stay for older adults residing in nursing homes at the end of life. J Am Geriatr Soc.

[23] Ontario Long Term Care Association. This is long term care 2016. Toronto, ON: Author. Retrieved September 18, 2017 from https://www.oltca.com/OLTCA/Documents/Reports/TILTC2016.pdf.

[24] Lawrence V, Samsi K, Murray J, Harari D, Banerjee S (2011). Dying well with dementia: qualitative examination of end-of-life care. Brit J Psychiat.

[25] Livingston G, Pitfield C, Morris J, Manela M, Lewis-Holmes E, Jacobs H (2012). Care at the end of life for people with dementia living in a care home: a qualitative study of staff experience and attitudes. Int J Geriatr Psych.

[26] Goddard C, Stewart F, Thompson G, Hall S (2013). Providing end-of-life care in care homes for older people: a qualitative study of the views of care home staff and community nurses. J Appl Gerontol.

[27] Marcella J, Kelley ML. “Death is part of the job” in long-term care homes: supporting direct care staff with their grief and bereavement. SAGE Open. 2015. 10.1177/2158244015573912.

[28] Kaasalainen S, Sussman T, Bui M, Akhtar-Danesh N, Laporte RD, McCleary L, O’Leary J (2017). What are the differences among occupational groups related to their palliative care-specific educational needs and intensity of interprofessional collaboration in long-term care homes?. BMC Palliat Care..

[29] Schulz M (2017). Taking the lead: Supporting staff in coping with grief and loss in dementia care. Health Manage Forum.

[30] Wickson-Griffiths A, Kaasalainen S, Brazil K, McAiney C, Crawshaw D, Turner M, Kelley ML (2015). Comfort care rounds: a staff capacity-building initiative in long-term care homes. J Gerontol Nurs.

[31] Sarabia-Cobo CM, Pérez V, de Lorena P, Nuñez MJ, Domínguez E (2016). Decisions at the end of life made by relatives of institutionalized patients with dementia. Appl Nurs Res.

[32] Sussman T, Kaasalainen S, Mintzberg S, Sinclair S, Young L, Ploeg J, Mc Kee M. Broadening the purview of comfort to improve palliative care practices in LTC. Can J Aging. 2017;36(3):306–17. 10.1017/S0714980817000253 28747236

[33] van Uden N, Van den Block L, van der Steen JT, Onwuteaka-Philipsen BD, Vandervoort A, Vander Stichele R, Deliens L (2013). Quality of dying of nursing home residents with dementia as judged by relatives. Int Psychogeriatr.

[34] Thompson Genevieve N., Roger Kerstin (2013). Understanding the needs of family caregivers of older adults dying with dementia. Palliative and Supportive Care.

[35] Moore K, Davis S, Gola A, Harrington J, Kupeli N, Vickerstaff V, King M, Leavet G, Nazareth I, Jones L, Sampson E (2017). Experiences of end of life amongst family carers of people with advanced dementia: longitudinal cohort study with mixed methods. BMC Geriatr.

[36] Thompson GN, McClement S, Menec V, Chochinov H (2012). Understanding bereaved family member’s dissatisfaction with end-of-life care in nursing homes. J Gerontol Nurs.

[37] Karascony S, Chang E, Johnson A, Good A, Edenborough M (2015). Measuring nursing assistants’ knowledge, skills and attitudes in a palliative approach: a literature review. Nurs Educ Today.

[38] Hansen L, Archbold PG, Stewart B, Westfall UB, Ganzini L (2005). Family caregivers making life-sustaining treatment decisions: Factors associated with role strain and ease. J Gerontol Nurs.

[39] Barnes S, Gardiner C, Gott M, Payne S, Chady B, Small N, Seamark D, Halpin D (2012). Enhancing patient-professional communication about end-of-life issues in life-limiting conditions: a critical review of the literature. J Pain Symptom Manag.

